# Predicting 3D moisture sorption behavior of materials from 1D investigations

**DOI:** 10.1038/s41598-020-74898-w

**Published:** 2020-10-20

**Authors:** Hom N. Sharma, Yunwei Sun, Elizabeth A. Glascoe

**Affiliations:** Lawrence Livermore National Laboratory, 7000 East Ave., Livermore, CA 94550 USA

**Keywords:** Chemical engineering, Characterization and analytical techniques, Chemical physics

## Abstract

Moisture in materials can be a source of future outgassing and exacerbate unwanted changes in physical and chemical properties. Here, we investigate the effect of sample size and shape on the moisture transport phenomena through a combined experimental and modeling approach. Several different materials varying in size and shape were investigated over a wide range of relative humidities (0–90%) and temperatures ($30{-}70^{\,\circ } \hbox {C}$) using gravimetric type dynamic vapor sorption (DVS). A dynamic triple-mode sorption model, developed previously, was employed to describe the experimental results with good success; the model includes absorption, adsorption, pooling (clustering) of species, and molecular diffusion. Here we show that the full triple-mode sorption model is robust enough to predict the dynamic uptake and outgassing of 3-dimensional (3D) samples using parameters derived from quasi-1D samples. This successful demonstration on three different materials (filled polydimethylsiloxane (PDMS), unfilled PDMS, and ceramic inorganic composite) illustrates that the model is robust at describing the scale-independent physics and chemistry of moisture sorption and diffusion materials. This work demonstrates that while sorption mechanisms manifest in testing of all sample sizes, some of these mechanisms were so subtle that they were overlooked in our initial modeling and assessment, illustrating the importance of multi-scale experiments in the development of robust predictive capabilities. Our study also outlines the challenges and viable solutions for global optimization of a multi-parameter model. The ability to quantify moisture sorption and diffusion, independent of scale, using 1D lab-scale experiments enables prediction of long-term bulk materials behavior in real applications.

## Introduction

Hygrothermal aging of materials is a concern to a wide range of industries including the food, packaging, medicine, electronics, aerospace, photovoltaics, and construction industries^1–5^. Moisture transport, sorption, and outgassing can change the physical and chemical properties of materials^5–10^, altering their performance and the performance of other materials in close proximity. Ultimately, moisture may impact the functionality of an entire component. Understating the material specific moisture capacity and transport behavior of each material in a component is essential to any long-term service-life assessment^2,11^. Describing these behaviors in a model enables service-life predictions; a robust model should be scalable as material size and shape (e.g., thin film versus large blocks) can vary significantly depending on the component and application.

While many lab scale moisture sorption experiments are conducted using thin samples (i.e., one dimensional or 1D type), many real applications require materials with significantly large dimensions (i.e., bulk material or 3D type)^12–15^. For example, quantifying the moisture distribution in concrete structures, which are typically 3D geometries, is essential for damage assessment and lifetime estimation^4^. Testing materials extensively in a controlled lab setting using large samples may not be feasible or cost effective. In such scenarios, extrapolation of the material moisture sorption behavior from lab scale 1D geometry to actual shape/size (often 3D geometry) is required but raises several questions.

The first question is whether moisture transport and sorption are scale-dependent properties of materials. If the materials are identical at different size scales and the 1D is materially identical to a 3D sample, then the fundamental physics of diffusion should be scale independent. Similarly, the sorption properties should not change as a function of scale. Thus, while basic logic suggests that the neither should be scale dependent, it is possible that some of the mechanisms may be subtle and unmeasurable in 1D samples but manifest more prominently in testing of 3D samples. The second question is whether a model can be developed, based on experiments from 1D samples, with enough fidelity to describe the mechanisms and predict these phenomena in 3D or bulk geometry samples. The outcome of these questions is crucial for capturing total moisture uptake capacity and subsequent outgassing and eventually the system-level performance and service-life.

Historically, moisture sorption and shelf life estimations of a material used the classic combination of the Fickian type model for the diffusion rate and various isotherm models (i.e., single mode and dual mode) to quantify the equilibrium moisture uptake^16–19^. Popular sorption models include Henry’s and Langmuir models, in single-mode or dual-mode formulations^20–24^, or one of several mathematical models including BET^25^, GAB^26^, Halsey^27^, Peleg^28^. These functional models are useful when the interest is in equilibrium sorption behavior, or when the scope of sorption is limited to a lower water-activity window. In reality, the moisture sorption behavior is a dynamic process and the aforementioned equilibrium models cannot adequately represent the actual behavior in a wide range of water activities^29–31^. Such discrepancies lead to large errors in the prediction of long term behavior (for example, shelf life estimation of food and medicinal products)^32^. Furthermore, bulk material properties in dynamic environments will deviate from the laboratory-based characterization of thin or small samples^33–35^. Without a unified dynamic multi-mode modeling approach, an accurate estimation of inherently dynamic materials–moisture interactions will be hindered..

Previously, a triple-mode sorption model was derived that includes a dynamic-Langmuir model, and equilibrium Henry’s and pooling models coupled to a Fickian diffusion model^36–38^. The dynamic triple-mode model describes the dynamic uptake of moisture in 1D samples exceptionally well but was never tested against 3D sample data.

Here we investigate the moisture sorption and diffusion behavior of materials over a range of sizes to bridge the gap between 1D and 3D scales. To our knowledge, there are few publications where sample thickness is systematically varied from 1D to 3D^39,40^. Our experiments span a range of sample thicknesses, humidities, and temperatures to reveal subtle changes in the data as measured via gravimetric analysis. Our study indicates that the phenomena exist in the thinnest samples but are difficult to definitively resolve until sample thickness is increased substantially. Using the dynamic triple-mode sorption model, we were able to simulate and match experiments on 3D samples using parameters derived from 1D experiments. However, optimization of the dynamic triple-mode model is difficult, and our results demonstrate that small, subtle features in the 1D-sample data may be easily overlooked and un-accounted for in the model parameters but may propagate to significant errors in the 3D-sample experiments. This paper demonstrates the consistency of sorption mechanisms over varying sample sizes, and the versatility and robustness of the dynamic triple mode model to describe these mechanisms. However, we also demonstrate that parameter calibration of the model is difficult and multiple sample shapes and sizes will increase the accuracy of the calibration and increase one’s confidence in predictions of other sample sizes and shapes, thus enabling accurate simulations of real-world shapes and sizes.

**Figure 1:**
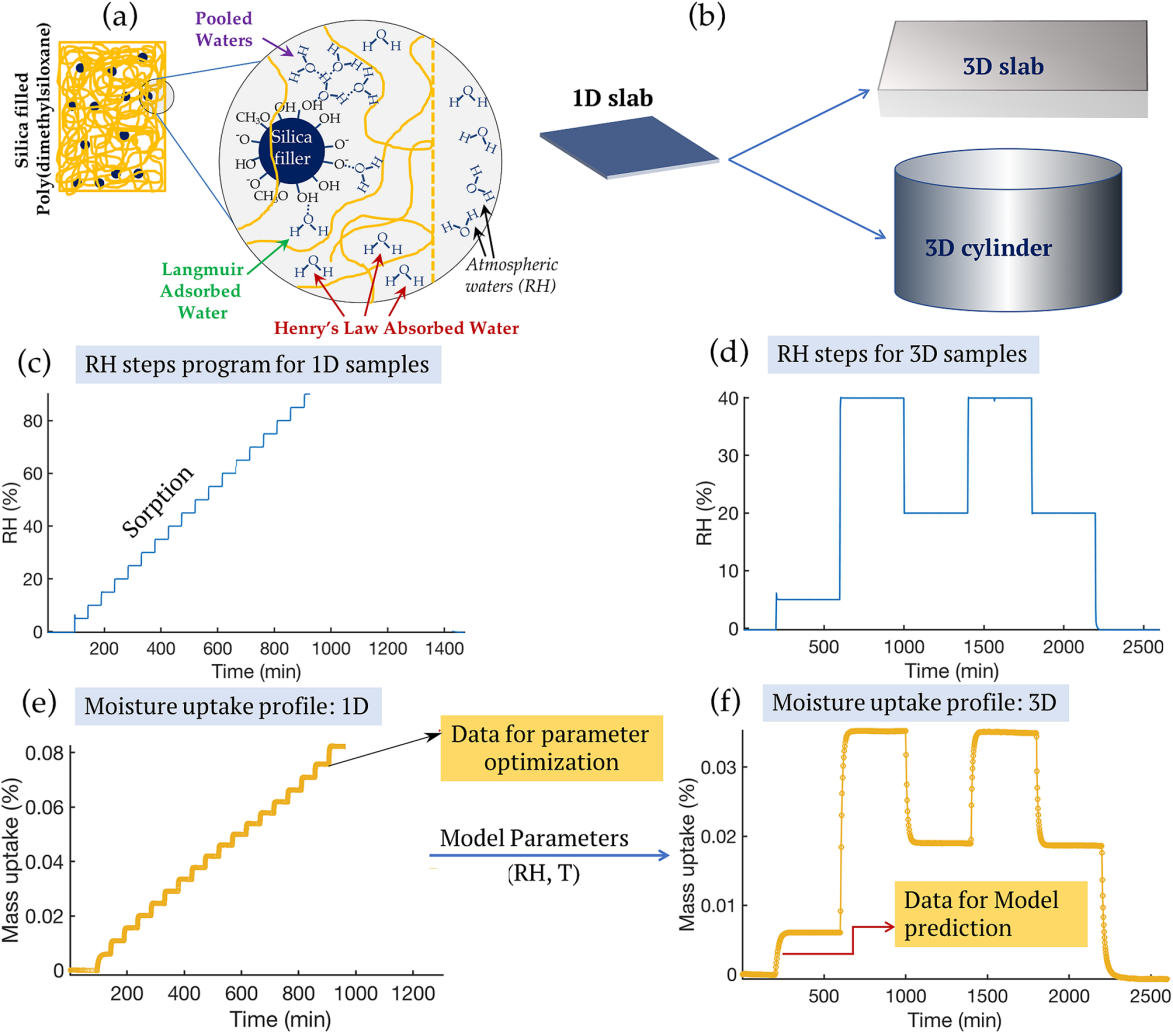
Panel (**a**): schematics showing the various modes (Langmuir, Henry’s, pooling) of vapor sorption in an example material, panel (**b**): schematics showing the shape/dimensions in 1D and 3D samples, panel (**c**): typical isotherm-experimental program for sorption and desorption characterization and model parametrization (relative humidity steps = 5–10% RH, duration of experiments will vary), panel (**d**): typical step-experimental program for model validation using 3D samples (RH and duration will vary but all will be large steps in humidity), panel (**e**): moisture uptake profile (in wt%) of a 1D sample resulting from the relative humidity program based on panel (**c**), and panel (**f**): moisture uptake by a 3D sample from the relative humidity program in panel (**d**).

This work involves a combined experimental and modeling approach to investigate sorption and diffusion phenomena in materials. Figure 1 shows the overall approach implemented in this study. Panel a shows the typical mesoporous material (i.e., silica filled polydimethylsiloxane here) where three different sorption modes (i.e., Henry’s, Langmuir, and pooling) are considered for the modeling. Samples are quasi-1D sheets or 3D samples in either blocks or right cylinders (see Fig. 1b); quasi-1D samples are hereafter referred to simply as 1D samples. Dynamic vapor sorption (gravimetric type) isotherm-experiments are conducted over a wide range of water activities to quantify the transient moisture-material interactions in 1D samples (Fig. 1c); these data are used to parameterize the model. Step-experiments (Fig. 1d) on 3D samples are used to validate the models and test its robustness against 3D samples and large humidity steps. Panels e and f (in Fig. 1) correspond to the sample weight change (due to moisture uptake and outgassing) when the samples are subjected to the RH environment as shown in panel c and d respectively. Details on the experimental setup, sample dimensions, materials, and model are discussed in the Method section.

## Results

### Continuum scale modeling using dynamic experiments

#### Sorption–diffusion model calibration

The dynamic-triple-mode sorption diffusion model is a continuum scale model consisting of three sorption modes (i.e., Henry’s, Langmuir, and pooling) and Fickian diffusion. The model is described in the method section and previous publications^36–38^ and allows for the mechanistic interpretation of experimental data.

Calibrating the nine parameters in this model requires a full isotherm at 5% humidity steps over a wide range of humidities and a multi-step optimization process to ensure the global minimum parameter set is identified. PSUADE^41,42^ (uncertainty quantification code and sampling-based search) is utilized in the initial parameter calibration step to access and narrow down the parameter range. In this method, over 3000 parameter sets were generated spanning user defined parameter ranges, using the Latin Hyper Cube sampling technique^43^. Each parameter set is then evaluated and compared with experimental data to obtain the associated objective function. The PSUADE results can be used to evaluate the global parameter sensitivity through Sobol’^44^ sensitivity analysis. Surface plots using multiple parameters and the objective function show local and global minima and guide users to a starting range for each parameter, which is necessary for next step of optimization: Shuffled Complex Evolution (SCE).

While the sampling based analysis method, PSUADE, can provide a set of coarsely tuned parameters, the SCE^45^ optimization, which is based on stochastic method, is a good choice for obtaining more precise and accurate model parameters for each sample. The SCE program is a metaheuristic global optimization approach for solving the minimization function, which utilizes multiple simplexes started from random locations in a defined parameter space through competitive evolution, and complex shuffling. Typically, PSUADE and SCE optimizations are performed using the isotherm-experimental data at one temperature (e.g., Fig. 1c,e).

Verification and validation of the optimized parameter set involves three steps. First the parameters are reviewed to ensure that they are physically reasonable. For example, diffusivity parameters are often available in the literature for comparison, if the specified material does not have a diffusivity measurement in the literature, there are often literature values for similar materials. Inspection of the isotherm curve reveals some indications of where Langmuir starts and plateaus out and where pooling commences. Second, parameter sensitivity analysis is performed to make sure the optimized values represent the global minimum. Finally, comparison with parameters from other materials provides some indications of what is physically reasonably. The next step in validation is to simulate experiments that were not part of the parameterization. The best validation is simulating step-experiments (e.g. Fig. 1d,f) from 1D- or 3D-samples.

**Figure 2:**
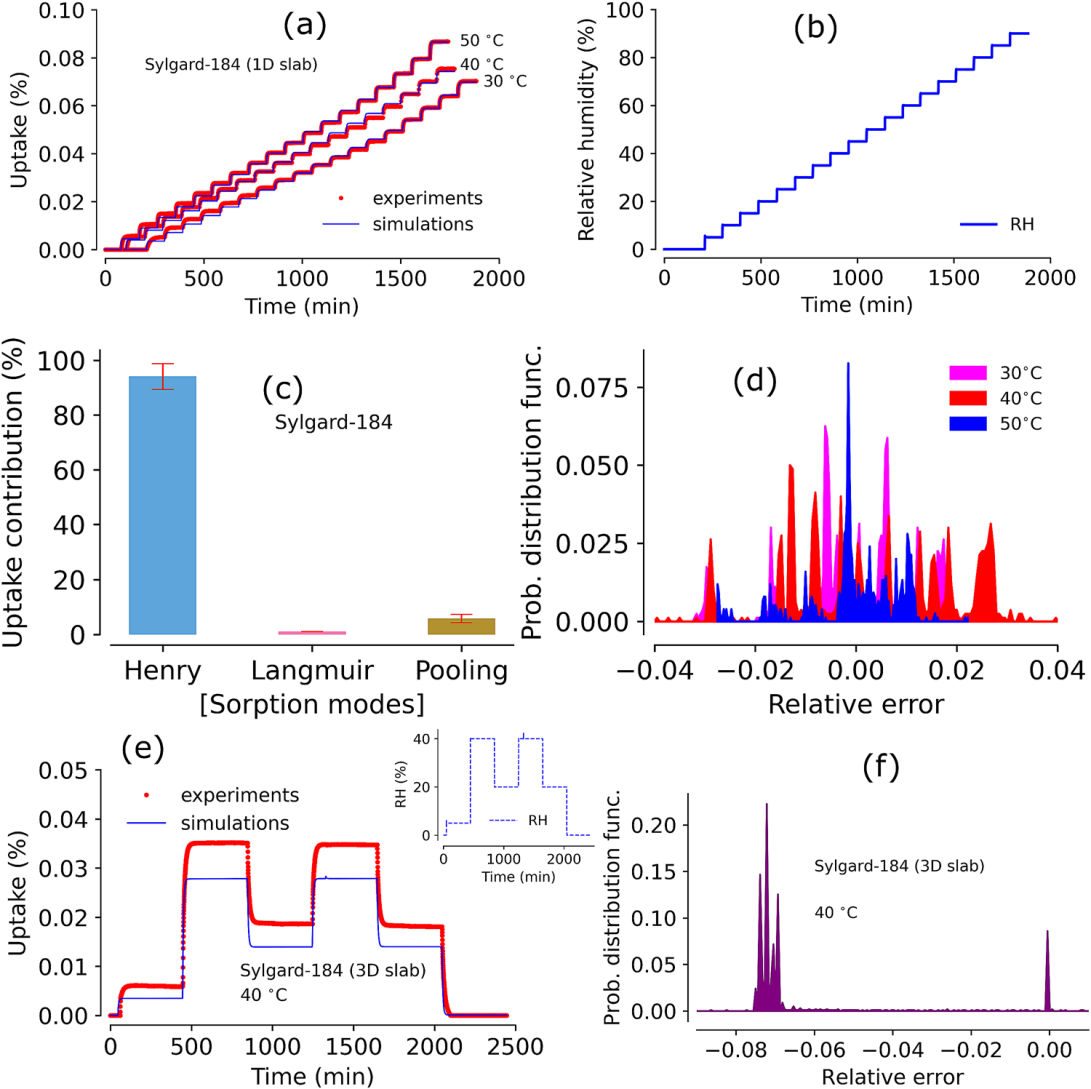
Panel (**a**): triple-mode sorption model simulations against Sylgard-184 1D data obtained at $30 {-} 50^{\,\circ } \hbox {C}$, panel (**b**): Relative humidity program set to obtain the moisture uptake gravimetric data in panel (**a**), panel (**c**): percent contributions by individual modes (with ${>} 90\%$ Henry’s mode) for total average moisture uptake in Sylgard-184, panel (**d**): relative error between experiments and simulations in Sylgard-184 1D slab samples at $30 {-} 50^{\,\circ } \hbox {C}$ , panel (**e**): model prediction against experimental data for a 3D sample of Sylgard-184 (RH steps used are given in inset plot) using parameters from panel (**a**), panel (**f**): relative error between experiments and simulations in Sylgard-184 3D slab sample.

#### Sorption diffusion modeling of Sylgard-184

Our triple-mode sorption diffusion model simulations were performed using previously optimized parameters on Sylgard-184 data^37^. Simulation profiles showed a good match against experimental data from thin 1D sample (see Fig. 2a). Relative humidity steps (in 5% increment) are shown in Fig. 2b. From simulations, the calculated average contribution from each mode (i.e., Langmuir, Henry’s, and pooling) is shown in Fig. 2c which suggests that the Henry’s mode is a major uptake mechanism with ${>}90\%$ of total moisture uptake. Results showed that the pooling mode contribution is very small and Langmuir mode contribution is insignificant or almost non-existent. In general, 95% of the error probability distribution falls within $\pm \, 4\%$ (as shown in Fig. 2d) suggesting a good match between experiments and simulations. Next, a new thicker slab (i.e., a 3D slab) was prepared (sample synthesized in a different lot) and step-RH (sequence as shown in the inset of Fig. 2e) experiments were conducted. The model simulations without further modification of parameters shows substantial mismatch compared to the experimental data (Fig. 2e). Error analysis shows two distinct group of probability distribution due to the mismatch (see Fig. 2f).

The mismatch between simulation and experiment in Fig. 2e is unacceptable; it will cast doubt on any simulation of 3D samples. There are three possible reasons for the mismatch, the first is that there may be phenomena that manifest in the 3D sample but do not appear in the 1D sample. These phenomena may be subtle or non-existent in the 1D experiments. A closer look into the experimental data and simulations revealed a few important sorption behaviors by Sylgard-184. Although the simulation profile in Fig. 2a matches the data well overall, there is a small discrepancy between simulation and experiments in the first few RH steps. We note that the Langmuir mode is typically active in the lowest RH steps until the surface sites are saturated. Moisture uptake in these low RH steps will often have a large uptake at each step, due to Langmuir sorption processes (further discussed in later sections). The discrepancy in the low RH region is attributed to an under-represented Langmuir mode that is not fully captured in the modeling parameters.

The second possible reason for the mismatch in Fig. 2e is the lot-to-lot variation of samples (i.e., variation introduced during production) between the 1D sample and the 3D sample. The third reason for the mismatch is that multi-parameter optimization can succumb to Hans Dreisch’s “equifinality” trap^46^ or may have some error due to the selection of goodness-of-fit method for objective function and convergence criteria. Since, the entire uptake curve is used to optimize the model parameters, the objective function is heavily skewed by the data and simulations at the higher RH regions. This shows the importance of selecting the right method to evaluate the goodness-of-fit and implementing checks for globally optimized parameters.

A second round of Sylgard-184 experiments were undertaken in order to explore the aforementioned uncertainties and determine if one of the three reasons listed above caused the mismatch in Fig. 2e. Samples with a wide range of thicknesses were prepared simultaneously from the same production lot. Each sample was exposed to the same humidity conditions in the DVS experiments to obtain an isotherm. The data obtained from each sorption experiment was utilized to parameterize the model and perform further analysis.

**Figure 3:**
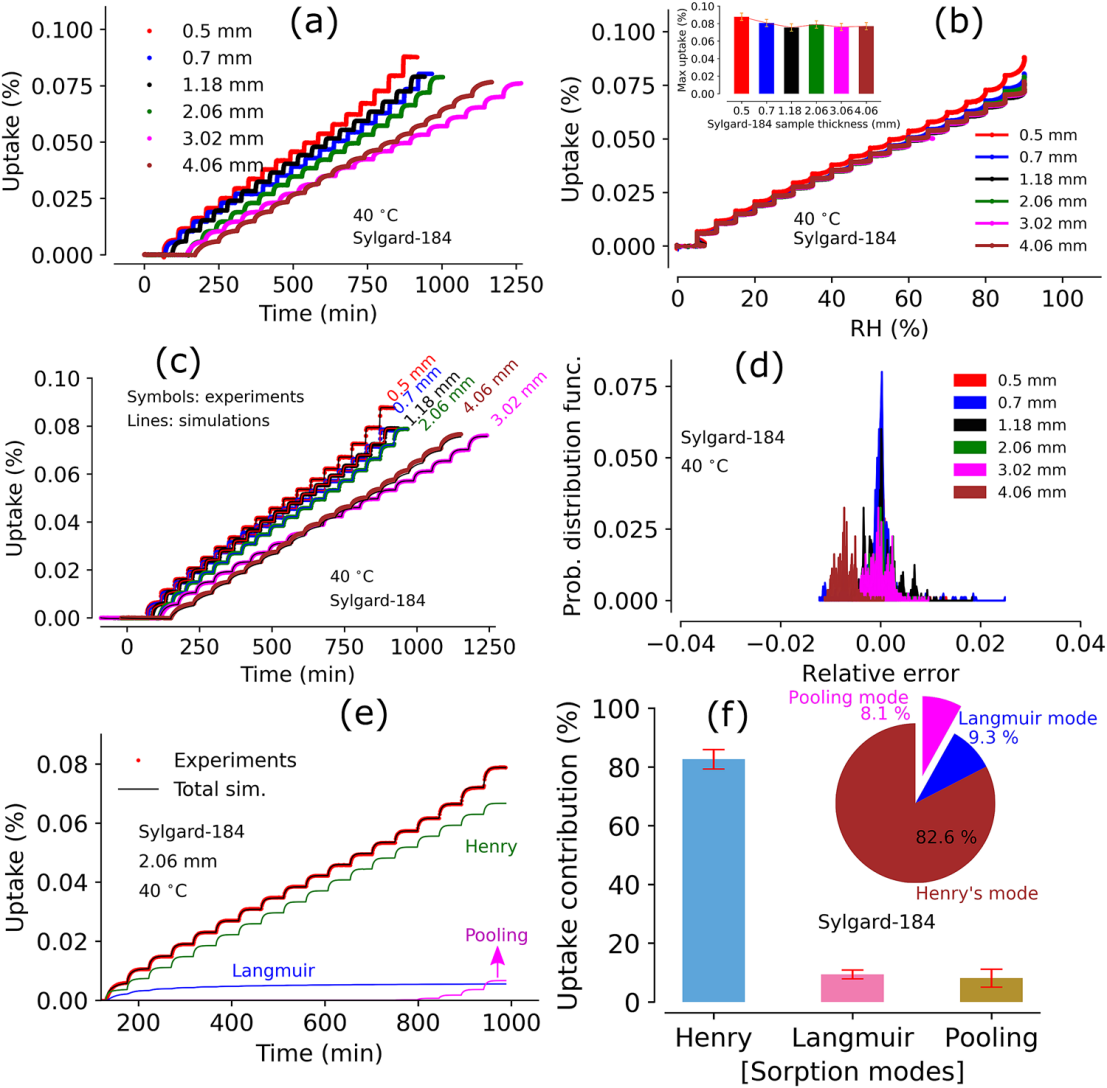
Panel (**a**): Moisture sorption profiles by sample with various thickness (${\sim }\, 0.5 {-} 4 \, \hbox {mm}$) of Sylgard-184 at $40^{\,\circ } \hbox {C}$. Panel (**b**): Moisture sorption and desorption (in %) versus relative humidity range (0–90%). Inset of panel (**b**) shows the maximum moisture uptake by each sample at 90% RH. Panel (**c**): triple-mode sorption model simulations using sorption data from each sample in Panel (**a**). Panel (**d**): relative error between experiments and simulations in Sylgard-184 various thickness slab samples at $40^{\,\circ } \hbox {C}$. Panel (**e**): individual contributions from three modes (Henry’s, Langmuir, and pooling) on total moisture uptake in a 2 mm thick sample of Sylgard-184. Panel (**f**): Average moisture uptake contributions (with $\pm \,1\sigma$) to total mass change from all modes in all samples considered. Inset figure of panel (**f**) shows the mode contributions for a representative 2 mm thick Sylgard-184 sample from panel (**e**).

#### Effect of sample thickness on moisture sorption in Sylgard-184

Figure 3a shows experimental uptake (%) vs time (in min) profiles of six different samples (from $\sim$ 0.5–4 mm thick) of Sylgard-184 at $40^{\,\circ } \hbox {C}$. Uptake curve shapes and durations differ slightly between the samples as the DVS program operates with a time window (i.e., minimum and maximum time for each RH step) rather than a fixed time step at each humidity step. Here, the instrument decides the hold time at a particular RH step within the set time interval based on 95% asymptote level for each uptake curve. Plotting uptake versus RH (see Fig. 3b) shows a fair comparison between the profiles of all the samples. Overall, uptake curves from various samples (with different thickness) exhibit similar uptake behavior. The inset figure (in Fig. 3b) shows the total uptake (%) by each samples at 90% RH which is $\sim$ 0.08%. Figure 3c shows the triple-mode sorption diffusion model simulations with SCE optimized parameters. Statistical analysis of error (error probability distribution) is within $\pm \, 3\%$ (see Fig. 3d) for all the samples and centered around zero, suggesting a good match between experiments and simulations.

Shown in Fig. 3e are the model and experimental results for the 2 mm thick Sylgard-184 sample. The contributions from all three modes and the total simulation curve are plotted in Fig. 3e. As discussed earlier, the major moisture sorption contribution is due to Henry’s mode (${>}80\%$). However, a smaller but significant contribution ($\sim$ 10%) from Langmuir mode can be clearly seen, which primarily occurs during initial RH steps. The pooling mode contribution is relatively small and only apparent above 85% relative humidity regions. This mode is not relevant for the system in low and mid-range humidity regions, where a dual-mode (i.e., Henry’s and Langmuir modes) model with only five parameters is sufficient. Figure 3f shows the contributions from all three modes averaged over all samples and the inset pie chart shows the same from a 2 mm thick sample.

**Figure 4:**
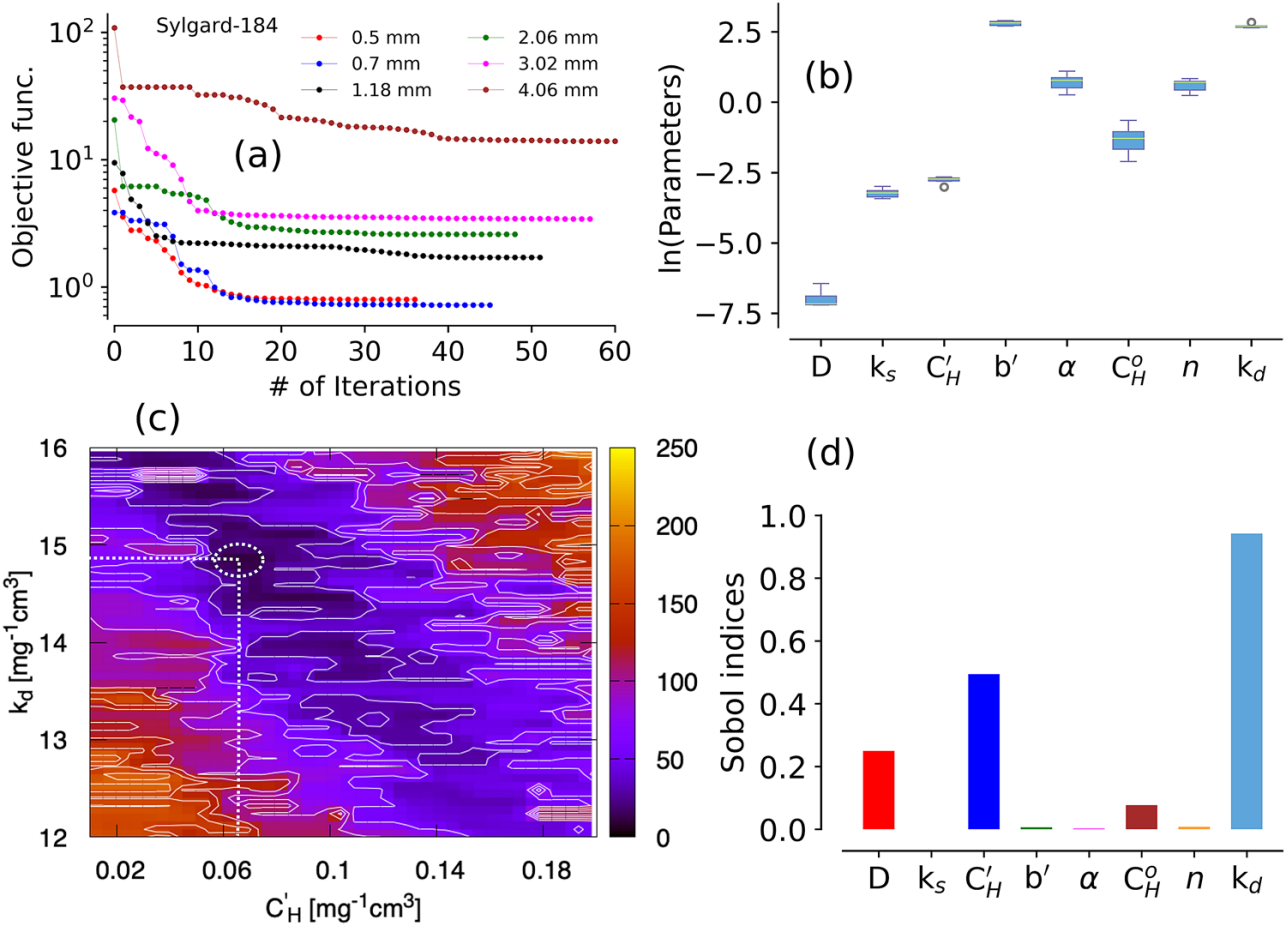
Panel (**a**): Objective function evolution during SCE optimization using experimental data from various samples of Sylgard-184 at $40^{\,\circ } \hbox {C}$; panel (**b**): box plot showing statistical distribution of model parameters obtained from SCE optimization using data from Sylgard-184 samples with various thickness ranging from 0.5 to 4 mm, panel (**c**): Surface plot showing Henry’s constant (${\hbox {k}}_d$) and Langmuir capacity ($C'_{H}$) variation in terms of objective function and the global minimum (at the intersection of the dotted lines). Color bar for the objective function values is shown on the right side of the plot; panel (**d**): Sobol’ total sensitivity index (TSI) for each model parameters obtained from PSUADE sampling based analysis.

The global minimization scheme using the SCE method ensures that the model parameters are well converged. The convergence profiles from several Sylgard-184 samples with multiple iterations are plotted in Fig. 4a. Typically, more than 30 iterations were required before the objective function meets the convergence criteria set for the optimization (see details in “Method” section). A box plot of the natural log of each model parameter (from all samples, $\sim$ 0.5 to 4 mm thick) is shown in Fig. 4b. One can see that in spite of the wide range of sample thicknesses, the parameter variability is quite narrow (model parameters are provided in the Supporting Information). This observation confirms that the sorption behavior does not vary with the sample thickness and it is a material property rather than shape or size dependent phenomena.

A surface plot constructed using the objective function variation between Langmuir capacity and Henry’s mode constant from sampling based simulations is shown in Fig. 4c. The minimum obtained from PSUADE analysis is consistent with the parameters obtained by SCE optimization method. Further, parameter sensitivity analysis was performed utilized sampling based PSUADE results by employing Sobol’ sensitivity method. Sobol’ sensitivity indices (shown in Fig. 4d) also confirmed that the dominant parameters are Henry’s mode constant (${\hbox {k}}_d$), Langmuir capacity (${\hbox {C}}'_H$), and diffusion coefficient (D) in a decreasing order. Sobol’ sensitivity is discussed in the “Methods” section.

**Figure 5:**
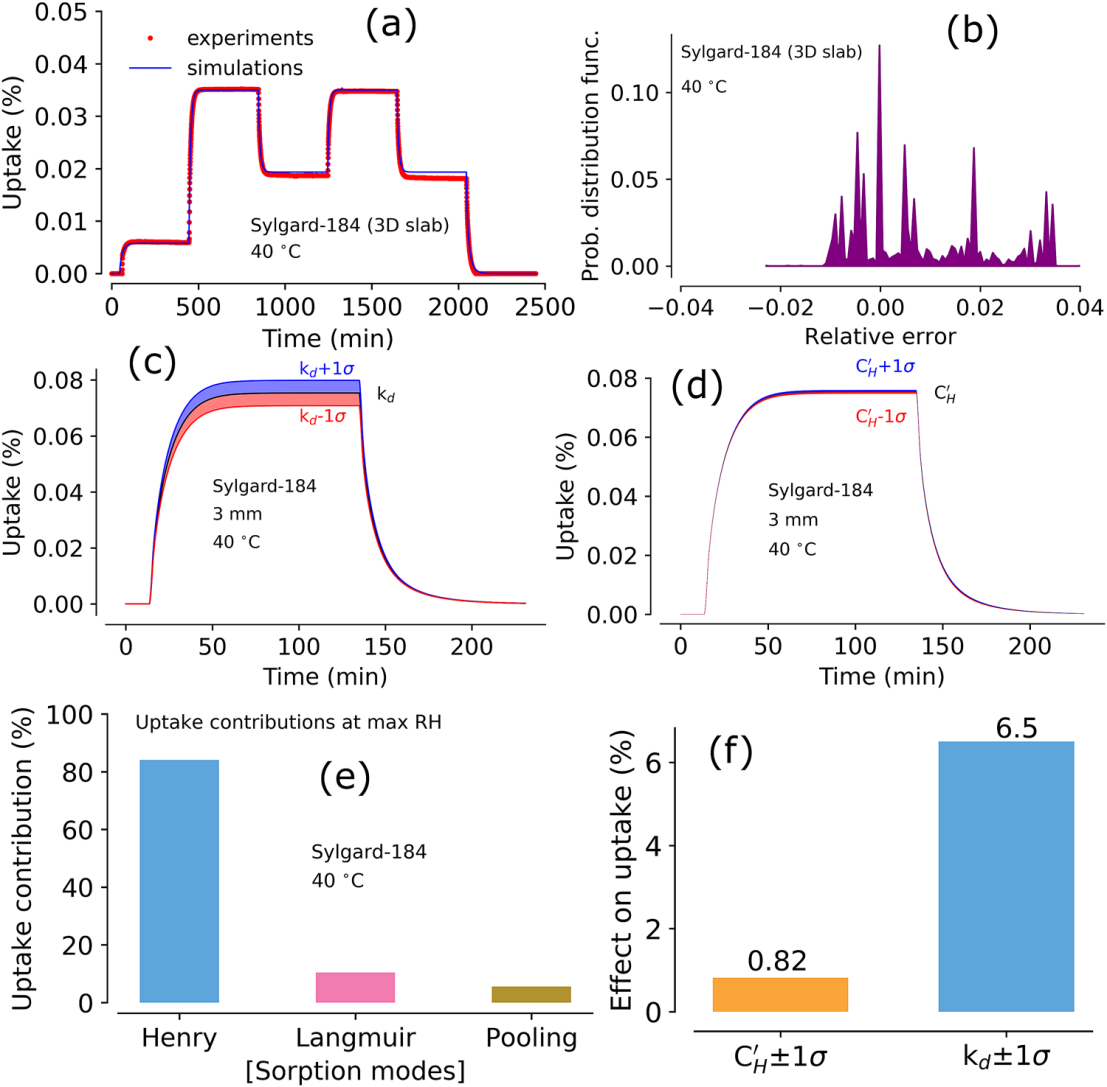
Panel (**a**): Model prediction of moisture sorption and outgassing for a 3D sample (Sylgard-184) at $40^{\,\circ } \hbox {C}$ using updated/optimized 1D parameters; panel (**b**): relative error between experiments and simulations in a 3D sample (Sylgard-184); panel (**c**): effect of ${\hbox {k}}_d \pm \, 1\sigma$ on total simulated moisture uptake (of the 3D sample) at $40^{\,\circ } \hbox {C}$ for a sequence of RH steps 0%–90%–0%; panel (**d**): effect of ${{\hbox {C}}'}_{H} \pm \, 1\sigma$ on total simulated moisture uptake (of the 3D sample) at $40^{\,\circ } \hbox {C}$ for a sequence of RH steps 0%–90%–0%; panel (**e**): The mode contributions from the simulations shown in the panel (**c**); panel (**e**): effect of change in parameter value (by $\pm \, 1\sigma$ of ${\hbox {k}}_d$ and ${{\hbox {C}}'}_{H}$) on total uptake as shown in panel (**d**) and panel (**c**), respectively.

### Predicting 3D Sylgard-184 moisture sorption

The optimized parameters from a 1D sample (from 0.7 mm sample) were used without further modifications to predict a random RH step moisture sorption profile of a thicker 3D sample (4 mm sample). Fig. 5a shows the experimental data and model predictions. The model prediction for the 3D sample moisture sorption and outgassing curve was a great match which is evident in the error probability distribution plot as shown in Fig. 5b; the distribution range was within ± 4 %.

Since the optimized parameters for all samples with different thicknesses show a range, albeit a narrow one, simulations were performed using a $1\sigma$ range of the most sensitive parameters ${\hbox {k}}_d$ and ${{\hbox {C}}'}_{H}$ (Henry’s constant and Langmuir capacity constant, respectively). The uptake profiles predicted using upper and lower end ${\hbox {k}}_d$ and ${{\hbox {C}}'}_{H}$ are displayed in Fig. 5c,d, respectively. The total uptake range varied slightly with ${\hbox {k}}_d$, which makes sense given the large capacity for moisture sorption via Henry’s mode. The uptake range was very narrow even when ${{\hbox {C}}'}_{H}$ was varied, which is probably due to the minimal overall sorption capacity of the Langmuir mode. Moreover, the simulations suggest that the total moisture uptake is $\sim$ 0.08 wt% at 90% RH as observed in 1D samples (see Fig. 3). This observation suggests that the 3D shape effect can be correctly captured with the parameters from 1D experiments. Figure 5e displays the percent contribution to the total moisture uptake from the simulations in panel c (Fig. 5). As expected, each mode contribution was similar to the one observed in a 1D sample (as shown in Fig. 3f). Furthermore, the effect of $1\sigma$ parameter range of ${\hbox {k}}_d$ and ${{\hbox {C}}'}_{H}$ to the total moisture uptake is shown in Fig. 5f. The variation in total moisture uptake predictions with $\pm \, 1\sigma$ of most sensitive parameters range was small with less than 6.5%. Further, we note that the external mass transfer resistance is negligible. However, the internal mass transfer resistance is captured using the calibrated diffusion coefficient. If the prediction of a 3D experiment using the parameters of 1D calibration agrees with results of 3D experiments, material can be assumed to be scale and dimension independent. Overall, the results show that prediction of bulk sorption behavior (for 3D type materials) can be made accurately with optimized parameters from 1D type experiments.

**Figure 6:**
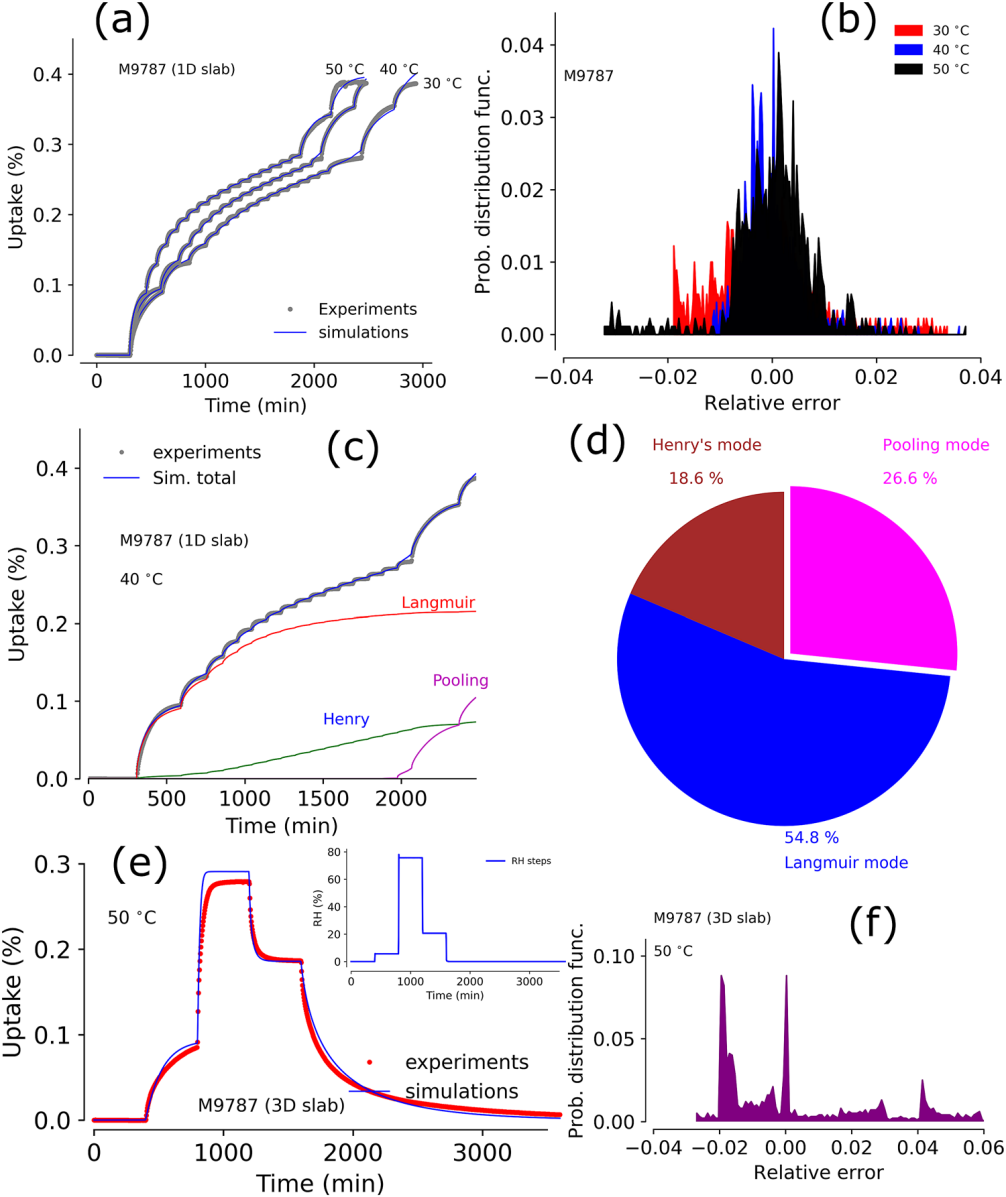
Panel (**a**): Model simulations of moisture sorption using SCE optimized parameters for a 1D sample (M9787) at 30, 40, and $50^{\,\circ } \hbox {C}$; panel (**b**): relative error between experiments and simulations in a 1D sample (M9787) at 30, 40, and $50^{\,\circ } \hbox {C}$; panel (**c**): Simulations showing each modes and dominant regions; panel (**d**): contributions from each modes to the total uptake in M9787 (1D slab) up to 90% RH conditions; panel (**e**): model predictions of moisture sorption and outgassing in a 3D sample of M9787 (inset shows the RH steps set during the experiment); panel (**f**): relative error between experiments and simulations in a 3D sample (M9787) at $50^{\,\circ } \hbox {C}$.

### Moisture sorption by a silica filled PDMS

Fumed silica filled PDMS polymer (M9787) showed distinctly different moisture sorption phenomena compared to Sylgard-184^36,47^. Experimental and model simulations results at 30, 40, and $50^{\,\circ } \hbox {C}$ and over a range of 0–90% RH are shown in Fig. 6a. The maximum moisture uptake (at 90% RH) was observed to be $\sim$ 0.4 wt%, which is $\sim$ 5 times larger than the Sylgard-184 moisture uptake at similar conditions. In general, the moisture sorption profile of M9787 clearly has three distinct regions. In the first region, at the lowest humidities ($\le 20\%$ RH), the moisture uptake steps are large and equilibrate relatively slowly. In the second region, 20–80% RH, the moisture uptake steps are small and rapid. In the third region, ${>}80\%$ RH, the uptake is dramatically different with large and slow uptake at each humidity step. These moisture uptake curves are distinctly different from the data for Sylgard-184 in Figs. 2a and 3a.

The M9787 data was used to calibrate a dynamic triple-mode model and one can see the simulation results overlaid on the experimental data in Fig. 6a. Error probability distributions at different temperatures, obtained from the triple-mode sorption model simulations of experimental profiles, are centered around zero and fall within ± 4% (see Fig. 6b). Moisture sorption contributions (at $40^{\,\circ } \hbox {C}$) by each modes from the model simulations are shown in Fig. 6c. Surprisingly, the contribution from Langmuir mode is very large and dominant. The Henry’s mode contribution is steady and significant. As expected, pooling mode was only present above 80% RH. Typically, Langmuir, Henry’s, and pooling mode contributions were $\sim$ 54.8%, $\sim$ 18.6%, and $\sim$ 26.6% of total moisture uptake by M9787 samples (as shown in Fig. 6d). Large Langmuir mode sorption implies that the material has large number of surface sites and is porous in structure. Presence of silica filler, hence the large surface sites, is responsible for such porosity in M9787. The mesoporous structure is also responsible for multi-layer formation and eventually clustering of water due to pore filling, which is source of pooling mode contribution.

Next, sequence type experiments were performed using a 3D sample of M9787. The sample was exposed to a sequence of RH steps (0%–5%–75%–20%–0%) as shown in the inset of Fig. 6e. Using the optimized parameters from the 1D sample data, moisture uptake and outgassing profiles were predicted using the 3D sample mesh thickness and compare with the experimental results from the 3D M9787 sample. Experimental data and model predicted uptake profiles are shown in Fig. 6e. Overall, our model prediction, without any parameter adjustment, was good and 95% of the error probability distribution falls within ± 5% (as shown in Fig. 6f). This validation of the model to simulate thicker samples is essential as it enables us to conduct experiments and optimize parameters using 1D samples and predict the 3D bulk material behavior with confidence. Conducting experiments with 3D samples can take substantially more time to get equilibrium data and may be constrained by the instrument limitations of sample size limits. Furthermore, actual 3D meshing and model simulations are costly (i.e., time and computationally), which may not always provide overwhelmingly more benefit over a cost-effective 1D model. Most importantly, the ability to accurately simulate data from 3D samples with modeling parameters from 1D-samples supports the hypothesis that the dynamic triple-mode model captures the moisture sorption and diffusion physics sufficiently well to apply beyond the data used for calibration.

**Figure 7:**
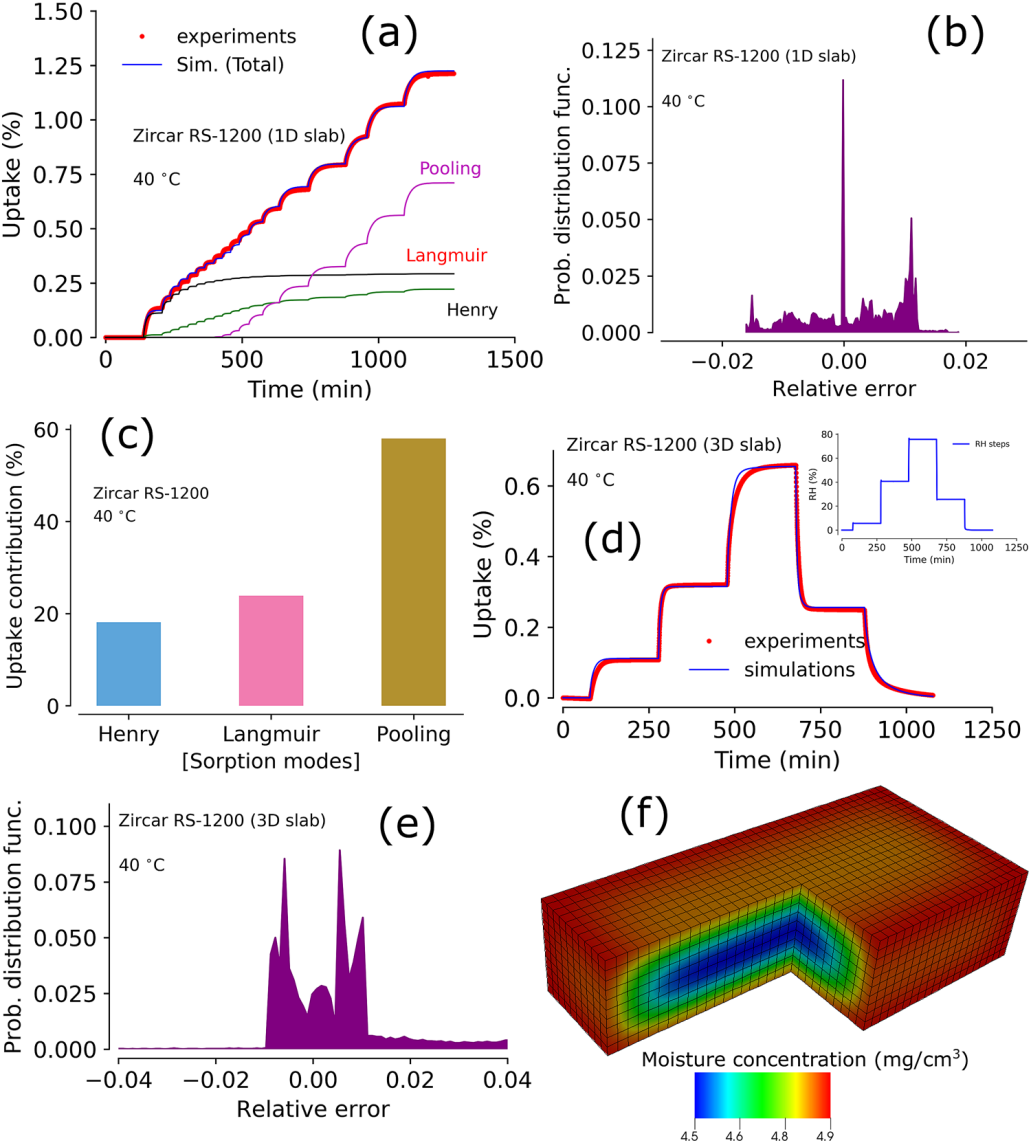
Panel (**a**): Comparison of experimental data and model simulations using the triple-mode sorption model for a 1D sample of Zircar RS-1200 at $40^{\,\circ } \hbox {C}$ (note this data was used to calibrate the model); panel (**b**): relative error between experiments and simulations in a 1D sample (Zircar RS-1200) at $40^{\,\circ } \hbox {C}$; panel (**c**): contribution of individual modes to the total uptake up to 90% RH conditions as shown in panel (**a**); panel (**d**): Comparison of experimental data and prediction of moisture sorption/outgassing from a 3D sample of Zircar RS-1200 at $50^{\,\circ } \hbox {C}$ using optimized parameters from 1D sample; panel (**e**): probability distribution of error between experiments and simulations in panel (**d**); snapshot of simulated moisture concentration profile (in $\text {mg}/{\text {cm}}^3$) in the 3D sample at the time frame of 500 min from the profile in panel (**d**).

**Figure 8:**
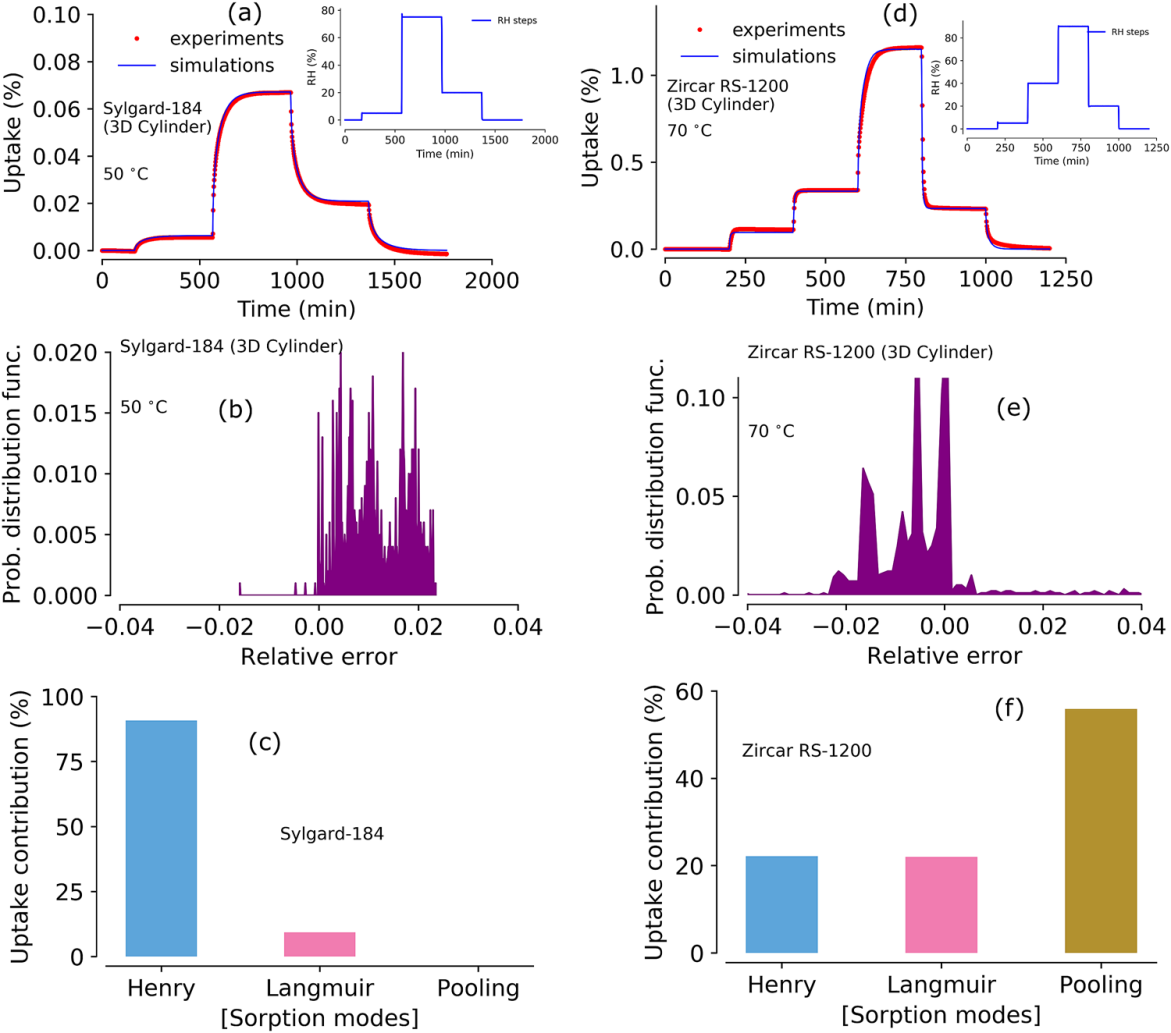
Experimental data and model prediction of moisture sorption and outgassing from cylindrical samples. Panel (**a**): experiments and model predictions of moisture sorption in cylindrical Sylgard-184 at $50^{\circ } \hbox {C}$ using optimized parameters from 1D sample triple-mode sorption model. Inset shows the RH steps used for the DVS experiments, panel (**b**): relative error between experiments and simulations in the 3D cylindrical Sylgard-184, panel (**c**): Individual moisture contributions calculated in 3D Sylgard-184 using triple-mode sorption model, panel (**d**): experiments and model predictions of moisture sorption in a 3D cylindrical Zircar RS-1200 sample at $70^{\,\circ } \hbox {C}$ using optimized parameters from 1D slab sample and triple-mode sorption model. Inset shows the RH steps used in the DVS experiments, panel (**e**): relative error between experiments and simulations in a 3D cylindrical sample (Zircar RS-1200) at $70^{\,\circ } \hbox {C}$, panel (**f**): Individual moisture contributions in 3D cylindrical Zircar RS-1200 calculated using triple-mode sorption model.

### Moisture sorption in ceramic material Zircar RS-1200

A ceramic material with alumina-silicate matrix, Zircar RS-1200, was considered to explore moisture sorption behavior using 1D and 3D type samples. Experimental dynamic moisture sorption profile and triple-mode sorption model simulations are shown in Fig. 7a. This data was used to calibrate the model and, overall, the model captures the sorption profile of the material. Error analysis showed that the model simulations are within ± 2% (see Fig. 7b) suggesting a good match. Unlike previous materials, distinct Langmuir and pooling modes can be seen in the figure. These two sorption modes are responsible for more than 80% of the moisture uptake by Zircar RS-1200 (see Fig. 7c). Langmuir sorption is large due to the presence of aluminosilicate matrix in the material, which provides large number of surface sites for water adsorption. Further, such aluminosilicate matrix creates mesoporosity in the material, making it a perfect candidate for clustering mechanism. The Langmuir sorption mode is substantial and attributed to a chemisorption type interaction, which can outgas moisture slowly for a long time during storage. Thus, Zircar RS-1200 may not be a good candidate for an application with moisture sensitive components due to its outgassing potential.

Shown in Fig. 7d are the experimental results of a dynamic sorption and outgassing experiment on a 3D sample exposed to a series of disparate RH-steps. Model predictions of this experiment were performed using the optimized modeling parameters from the1D-experiments. Comparison of the model predictions against the experimental data captures the entire profile with an error margin of ± 2% (see Fig. 7e). Figure 7f shows a snapshot of moisture concentration at a given time, where a gradient of moisture concentration can be seen. Eventually, the concentration of the moisture reaches the same level everywhere, which can be defined as an equilibrium uptake capacity for a given condition (temperature and RH). We note that the hysteresis can be seen during desorption at a higher RH range^37^. However, evaluating a specific contribution from such a phenomenon is not the scope of this work. Nonetheless, the hysteresis effect should be taken into consideration for applications involving elevated RH conditions.

### Prediction of shape effect on moisture sorption

Owing to our successful predictions of a 3D slab type sample results from 1D slab parameters, we extended our investigation of experiments and model simulations to 3D cylindrical samples. Here, Sylgard-184 and Zircar RS-1200 samples are considered to show the model flexibility going beyond the slab-type geometry. Figure 8 shows the model performance and analysis for the cylindrical samples. All the simulations parameters for 3D cylindrical samples are taken from optimized 1D slab results. Figure 8a shows the moisture uptake profile and model prediction for a 3D cylinder sample of Sylgard-184 at $50^\circ \hbox {C}$ (the RH steps used for the experiment are shown in the inset). Overall, the model prediction was good and relative error between experiments and simulations was within ± 3% (see Fig 8b). Contributions from the different sorption modes to overall moisture uptake by the sample are provided in Fig. 8c. We note that no pooling contribution was observed in the simulation; the pooling mode in Sylgard-184 is only active above 80% RH regions, whereas this experiment only goes to 75% RH. As expected, the most moisture uptake contribution comes from Henry’s mode.

Model performance was also tested against the experimental data obtained from a 3D cylindrical sample of ceramic Zircar RS-1200 at $70^\circ \hbox {C}$ as shown in Fig. 8d. Experimental RH conditions are shown in figure inset and highest set point goes above 80% RH. Overall the model performs well by capturing the entire moisture uptake and outgassing profile. Probability error distribution was within the range of ± 3–4% as shown in Fig. 8e. The contributions from each mode, shown in Fig. 8f, are similar to the slab sample, which was expected; most of the moisture uptake contribution comes from Langmuir and pooling modes (see Fig. 7c).

### Concluding remarks

Material-moisture interactions are essential in terms of material stability, physiochemical changes, and system lifetime predictions. A detailed understanding of such interactions at the mechanistic level is always desired for many system-level designs. This work broadly focuses on two aspects of the material-moisture interactions. First, we employ a combined experimental and triple-mode sorption diffusion modeling approach to understand and quantify the moisture sorption by various thin (1D or quasi-1D) type samples. Second, we predict the moisture sorption and outgassing by the bulk (or 3D type) material utilizing the information obtained from 1D type samples, which is more relevant to the practical applications. Three different types of materials (i.e., a PDMS Sylgard-184, silica filled M9787, and ceramic Zircar RS-1200) are chosen for the study, which show widely different interactions with moisture.

Our triple-mode sorption diffusion model (with Henry’s mode, Langmuir mode, and pooling mode) accurately captures the entire moisture interaction profiles from dry to 90% relative humidities. In some cases (for example, in Sylgard-184), special attention is required to obtain the model parameter global minimum when a sorption mode is very insignificant or small. This observation was evident from our systematic study by considering multiple samples with varying thickness, which revealed a small but essential Langmuir mode sorption at low relative humidities. In general, Sylgard-184 sorption mode is dominated by Henry’s mode with contributions ${>}80\%$ of the total moisture uptake at 90% RH. Unlike Sylgard-184, a silica filled PDMS (M9787) showed much different moisture interaction behavior with $\sim$ 55% total moisture contribution coming from Langmuir mode. This ‘hydrophilic’ tendency can be explained by the presence of silica filler in the M9787. The chemisorption type (i.e., Langmuir) interaction with silica is more likely to create long term outgassing problems in many systems with moisture sensitive units as this is harder to remove from the surface. Surprisingly, most of the moisture uptake contributions ($\sim$ 60%) in ceramic material Zircar RS-1200 are from pooling mode sorption, primarily due to the mesoporous aluminosilicate matrix. In all cases, the optimized model parameters obtained using experimental data from 1D samples were able to predict the moisture sorption/outgassing behavior of bulk (i.e., 3D type). Moreover, the 1D model parameters accurately predicted the shape-dependent moisture interaction in those materials. Finally, the work presented here may provide a framework for seamlessly taking a lab-scale sample testing information to design a system with bulk materials, as in many practical applications.

## Methods

### Experimental details

Experiments using all (1D and 3D type) samples were conducted using the IGAsorp^48^ instrument, designed by Hiden Isochema, which is equipped with a high resolution micro-balance.The measurement uncertainties include relative humidity (RH) accuracy of ± 1% (0–90%) with regulation accuracy of ± 0.1% RH, temperature measurement accuracy of $\pm \,0.1^{\,\circ } \hbox {C}$ with regulation accuracy of $\pm \,0.1^{\,\circ } \hbox {C}$, and weight resolution of $\pm \, 1 \, \upmu \hbox {g}$ (maximum sample size of 5 g). Due to stringent calibration and certification of microbalance, the uncertainty is not significant for practical purposes. We have tested the reproducibility of the data by performing experiments multiple times under the same conditions (temperature/RH). We note that the data acquired from the instrument are reproducible and error margin is less than 1%. Further details are provided in the Supporting Information. Details on the equipment and experimental setup can be found in prior publications^37,38^. Herein, 3 different types of materials (i.e., Sylgard-184, Zircar RS-1200, and silica filled PDMS M9787) were used in this study. Sylgard-184 is a commercially available polydimethylsiloxane elastomer, sold by the Dow chemical company, that was prepared in-house following the mixing ratio provided by the manufacturer. Zircar RS-1200 composite consists of strong reinforcing fibers tightly bound in an alumina ceramic matrix composition: ${\sim } \, 82\% \, {\hbox {Al}}_2 {\hbox {O}}_3$, ${\sim } \, 12\% \, {\hbox {SiO}}_2$, and $\sim$ 6% other metal oxides, density = 2.16 g/cc and porosity = 31%) and designed for use as thermo-mechanical and electrical insulation. Our samples were obtained from ZIRCAR Refractory Composites, Inc., and machined to the appropriate sample dimensions without the use of water or solvents. M9787 is a silica-filled silicone, which is formulated in-house, contains 21.6% fumed silica filler (Cab-O-Sil-M-7D), 4% precipitated silica filler (Hi-Sil 233), 67.6% polysiloxane gumstock, and 6.8% processing aid^2^.

Typically, thin slab type (1D) samples were used to get the complete isotherm (from dry to 90% RH with 5–10% RH steps) and thicker (3D) samples were used to run random RH step (for example: dry—10% RH—90% RH—20% RH—dry) experiments. Generally, a single sample for a material (except for the experiments to study sample thickness effect) was used for various the 1D-experiments (i.e., at different temperatures) used for the model parameter estimations reported here. Sample dimensions of Sylgard-184 are as follows: typical 1D samples with length = 3.0 cm, width = 2.1 cm, and thickness = 0.5–4.0 mm; for 3D cylinder: radius = 0.64 cm, height = 1.15 cm. Sylgard-184 sample density was 0.982 g/cc. Sample dimensions of M9787 are as follows: for 1D, length = 1.99 cm, width = 1.94 cm, and thickness = 0.185 cm; for 3D slab, length = 1.95 cm, width = 1.93 cm, and thickness = 0.42 cm. Sample dimensions of Zircar RS-1200 are as follows: for 1D slab, length = 2.65 cm, width = 1.07 cm, and thickness = 0.33 cm; for 3D slab, length = 1.51 cm, width = 1.212 cm, and thickness = 0.56 cm; for 3D cylinder, radius = 0.5 cm, height = 1.0 cm. M9787 sample density was 1.145 g/cc. Furthermore, we note that the detailed morphological characterization was not the scope of this study.

Before starting an experiment, each sample was preconditioned (dried) under dry ${\hbox {N}}_2$ gas flow of 250 mL/min in the instrument chamber for many hours to establish a dry weight reference and served as a baseline for wt% moisture uptake calculations.Drying time depends upon the sample (material) type and size. Typically, thinner samples (1D type) require shorter drying time (for example, 10–12 h for Sylgard-184) and thicker samples (3D type) need a longer time (for example, 3–4 days for Zircar RS-1200 3D slab) to achieve the baseline weight. The drying step of the sample was performed before the start of the experiment.After we achieve the rate of change of mass ${<}\,0.001 \, \hbox {mg/min}$ in the drying curve, we start the isotherm experiment. For the actual experiments (shown in the “Results” section), we program the instrument to collect the dry state (0 % RH) data for few hours (3–4 h) to make sure we have the accurate baseline point. Typically, the drying temperature is the same as the experimental temperature. IGAsorp performs real-time prediction of the sorption profile to determine the appropriate time to move to the next RH level. The model criterion is based on an exponential linear driving force fitting of the data. Further details are provided in the Supporting Information. Our typical experimental range was 0–90% RH for humidity and $25 {-} 70^{\,\circ } \hbox {C}$ for temperature. Relative humidity can be converted to water activity (${\hbox {a}}_w$) by diving the RH by 100 (i.e., ${\hbox {a}}_w = \hbox {RH}(\%)/100$, range = 0–1). Moisture uptake (in %) is defined as,

1 $$\begin{aligned} u = \frac{m-m_{\mathrm{0}}}{m_{\mathrm{0}}} \times \,100 \end{aligned}$$

where $m_{\mathrm{0}}$, *m*, and *u* are the initial dry mass, instantaneous moist mass, and the percentage mass uptake, respectively.

### Diffusion–sorption model

The mass balance equation of vapor sorption and diffusion in a porous material is^36–38^

2 $$\begin{aligned} \frac{\partial C}{\partial t}= \nabla \cdot \left( D\nabla C \right) +{{{{\mathscr {S}}}}}_{\mathrm{k}} + {{{\mathscr {S}}}}_{\mathrm{e}} \end{aligned}$$

where *C* [${\text {mg g}}^{-1}$] is the mobile gas concentration in terms of sample bulk mass, *D* [${\text {cm}}^2 \, {\text {min}}^{-1}$] is diffusion coefficient, *t* [min] is the time, ${{{\mathscr {S}}}}_{\mathrm{k}}$ and ${{{\mathscr {S}}}}_{\mathrm{e}}$ represent kinetic and equilibrium sorption operators. The total sorption is composed of kinetic Langmuir adsorption, Henry’s absorption and pooling sorption in equilibrium^36–38^

3 $$\begin{aligned} {{{\mathscr {S}}}} = {{{\mathscr {S}}}}_{\mathrm{k}} + {{{\mathscr {S}}}}_{\mathrm{e}} = - \frac{\mathrm {d}C_{\mathrm{L}}}{\mathrm {d}t} - \frac{\mathrm {d}\left( C_{\mathrm{H}}+C_{\mathrm{P}}\right) }{\mathrm {d}t}. \end{aligned}$$

where $C_{\mathrm{L}}$, $C_{\mathrm{H}}$, and $C_{\mathrm{P}}$ [${\text {mg g}}^{-1}$] are the mass concentrations, respectively in Langmuir, Henry’s, and pooling modes, and measured in sorbed vapor mass per unit bulk mass of a material. Henry’s mode concentration, $C_{\mathrm{H}}$ can be directly treated as a mobile concentration due to its linear dependence of vapor concentration in equilibrium

4 $$\begin{aligned} C_{\mathrm{H}}= k'_{\mathrm{d}} \ C =\frac{k'_{\mathrm{d}} \ \phi }{\rho _{\mathrm{b}}} \ c = k_{\mathrm{d}} \ c, \quad k_{\mathrm{d}}=k'_{\mathrm{d}} \frac{\phi }{\rho _{\mathrm{b}}} \end{aligned}$$

in which $k_{\mathrm{d}}$ [${\text {cm}}^3 \, {\hbox {g}}^{-1}$] is the Henry’s law constant used in simulations, $k'_{\mathrm{d}}$ [–] is dimensionless Henry’s constant, $\phi$ [–] is the porosity (the fraction of pore volume over bulk volume), $\rho _{\mathrm{b}}$ [${\text {g cm}}^{-3}$] is the bulk density, *c* [${\text {mg cm}}^{-3}$] represents gas-phase concentration and is equivalent to *C* with a different definition. Assuming that porosity ($\phi$) and bulk density ($\rho _{\mathrm{b}}$) of porous materials are not altered by vapor diffusion and sorption, both parameters are implicitly lumped into Henry’s absorption constant, $k_{\mathrm{d}}$. Then, the mass balance equation can be expressed in terms of $C_{\mathrm{H}}$

5 $$\begin{aligned} \frac{\partial C_{\mathrm{H}}}{\partial t} = \nabla \cdot \left( D_{\mathrm{e}}\nabla C_{\mathrm{H}}\right) \overbrace{\underbrace{ - k_{\mathrm{a}} \ S \ C_{\mathrm{H}} + k_{\mathrm{s}} \ C_{\mathrm{L}}}_{\mathrm{Langmuir}} \underbrace{ - k_{\mathrm{p}}\frac{\mathrm {d}C_{\mathrm{P}}}{\mathrm {d}t}}_{\mathrm{Pooling}}}^{{{{\mathscr {S}}}}(C_{\mathrm{H}})} \end{aligned}$$

where $D_{\mathrm{e}} = D/(1+k'_{\mathrm{d}})$ [${\text {cm}}^2 \, {\text {min}}^{-1}$] is the effective diffusion coefficient, $k_{\mathrm{a}}$ [${\text {g mg}}^{-1} \, {\text {min}}^{-1}$] and $k_{\mathrm{s}}$ [${\text {min}}^{-1}$] are adsorption and desorption rates, respectively, $S = C'_{\mathrm{H}}-C_{\mathrm{L}}$ [${\text {mg g}}^{-1}$] is the empty Langmuir site concentration, $C'_{\mathrm{H}}$ [${\text {mg g}}^{-1}$] is the Langmuir capacity, and $k_{\mathrm{p}}$ [–] is the pooling constant. The kinetic Langmuir adsorption is expressed

6 $$\begin{aligned} \begin{aligned} \frac{\mathrm {d}C_{\mathrm{H}}}{\mathrm {d}t}&= -k_{\mathrm{a}} \ S \ C_{\mathrm{H}} +k_{\mathrm{s}} \ C_{\mathrm{L}}\\ \frac{\mathrm {d}C_{\mathrm{L}}}{\mathrm {d}t}&\approx k_{\mathrm{a}} \ S \ C_{\mathrm{H}} - k_{\mathrm{s}} \ C_{\mathrm{L}}. \end{aligned} \end{aligned}$$

Equation (5) can also be expressed in terms of *c*

7 $$\begin{aligned} \frac{\partial c}{\partial t} = \nabla \cdot \left( D_{\mathrm{e}}\nabla c\right) - k_{\mathrm{a}} \ S \ c + \frac{k_{\mathrm{s}}}{k_{\mathrm{d}}} \ C_{\mathrm{L}} - \frac{k_{\mathrm{p}}}{k_{\mathrm{d}}} \ \frac{\mathrm {d}C_{\mathrm{P}}}{\mathrm {d}t} \end{aligned}$$

with ODEs of Langmuir adsorption kinetics

8 $$\begin{aligned} \begin{aligned} \frac{\mathrm {d}c}{\mathrm {d}t}&= -k_{\mathrm{a}} \ S \ c +\frac{k_{\mathrm{s}}}{k_{\mathrm{d}}} \ C_{\mathrm{L}}\\ \frac{\mathrm {d}C_{\mathrm{L}}}{\mathrm {d}t}&\approx k_{\mathrm{a}}k_{\mathrm{d}} \ S \ c - k_{\mathrm{s}} \ C_{\mathrm{L}}. \end{aligned} \end{aligned}$$

As observed in experiments^36^, pooling sorption occurs only when *c* or $C_{\mathrm{H}}$ reaches a certain concentration ($C^0_{\mathrm{H}}$ or $c^0_{\mathrm{H}}$) and expressed in an isotherm form

9 $$\begin{aligned} C_{\mathrm{P}} = \alpha \,{{{\mathscr {H}}}}(C - C^0_{\mathrm{H}}) \cdot \left( C_{\mathrm{H}}-C^0_{\mathrm{H}}\right) ^n \end{aligned}$$

where $\alpha$ is a pooling constant, ${{{\mathscr {H}}}}$ is Heaviside function, and $C^0_{\mathrm{H}}$ [${\text {mg g}}^{-1}$] is pooling threshold concentration. Considering scaling issue in a power-law function, Eq. (9) is further revised in the format of Oswin sorption isotherm^49^

10 $$\begin{aligned} C_{\mathrm{P}} = \alpha \,{{{\mathscr {H}}}}(C_{\mathrm{H}} - C^0_{\mathrm{H}}) \cdot \left( \frac{C_{\mathrm{H}} - C^0_{\mathrm{H}}}{C_{\mathrm{m}}+C^0_{\mathrm{H}}-C_{\mathrm{H}}}\right) ^n \end{aligned}$$

where $C_{\mathrm{m}}$ [${\text {mg g}}^{-1}$] is the maximum Henry’s concentration. To account for a decreased effective diffusion once pooling begins, a reduced tortuosity^36^ parameter $\tau$ was introduced at the point at which pooling started; below this point the tortuosity was 1.0. The effective diffusivity can be calculated as,

11 $$\begin{aligned} D = D_{o} \tau \end{aligned}$$

where *D* is effective diffusion coefficient, $D_{o}$ is molecular-weight-dependent diffusivity, and $\tau$ is medium-specific tortuosity, which is a measure of the connectivity of pores and defined as the chord-arc ratio (ratio of the straight distance to the integrated length of the tortuous pathway).

### Parameters estimation and optimization

Model parameters are estimated using the uncertainty quantification code PSUADE^41,42^ and calibrated using the shuffled complex evolution (SCE) method^45^. First, a sampling based non-intrusive Latin Hypercube (LH) sampling method^43^ is used to generate a large number of sample points; sufficiently large to represent the parametric space. Each ‘sample point’ consists of a vector of all parameters (i.e., *D*, $k_{\mathrm{s}}$, $C'_{\mathrm{H}}$, $b^\prime$, $C^0_{H}$, $\alpha$, *n*, $\tau$ and $k_{\mathrm{d}}$) in our model. To be consistent with equilibrium Langmuir formulation, we use the Langmuir affinity constant $b'= k_{\mathrm{a}}/ k_{\mathrm{s}}$ [${\hbox {mg}}^{-1} \hbox {g}$] instead of $k_{\mathrm{a}}$ in our parameter calibration. Then, each sample point is used to parameterize the model and the corresponding objective function is computed. Sample points resulting the smallest minimization function are chosen to be the candidates for the parameter optimization using the SCE^50^ method implemented in PSUADE^51^ with lower and upper bounds of all parameters defined. The objective function used for model calibration is:

12 $$\begin{aligned} f = \int _{t} |m(t) -{\hat{m}}(t)|dt \end{aligned}$$

in which *m* and ${\hat{m}}$ are the experimental and simulated mass uptake, respectively. The model simulated mass uptake is calculated as:

13 $$\begin{aligned} {\hat{m}} = \rho _{\mathrm{b}}\; A_{0} \int _{0}^{L} (C_{\mathrm{H}} + C_{\mathrm{L}} + C_{\mathrm{P}} )\;dx, \end{aligned}$$

where $\rho _{\mathrm{b}}$ and $A_{0}$ are the bulk sample density and sample area, respectively. SCE in PSUADE is used to find the best fit of model to experimental data with a set of best fit parameters. Our SCE optimization convergence criterion was set to $1 \times 10^{-5}$ for the relative change in the objective function. Once the convergence criterion has been met, the optimization is set to complete and the final parameters are obtained. Our model parameters are set to be accurate within the error margin of 0.01%.

Global sensitivity analyses are conducted by using the Sobol’ method^44^. The method measures contribution from each uncertain input to the variance of the output of interest. The total sensitivity index (TSI) of the output is calculated as the sum of all sensitivity indices, including all interactive effects. Further details can be found elsewhere^36,52^.

## Supplementary information

Supplementary Information.

## Data Availability

The data that support the findings (experimental and modeling results) of this study are available from the corresponding author upon reasonable request.
