# Supplementary material for: Predicting 3D moisture sorption behavior of materials from 1D investigations
Source: Sci Rep. 2020 Oct 20;10:17852. doi: 10.1038/s41598-020-74898-w (PMC7576181; doi:10.1038/s41598-020-74898-w)
Supplement: Supplementary file 1 — Supplementary Information. [file 41598_2020_74898_MOESM1_ESM.pdf]

Supplementary information for

Predicting 3D Moisture Sorption Behavior of Materials from 1D Investigations

Hom N. Sharma[1], Yunwei Sun[1], and Elizabeth A. Glascoe[1,*]

[1]Lawrence Livermore National Laboratory, 7000 East Ave., Livermore, California 94550, United States

[*] glascoe2@llnl.gov

S1. SCE optimized parameters for Sylgard-184 samples

Samples of six different thicknesses were considered in this study. Parameters are listed here for the experiments conducted at 40 °C.

Parameters	Sym	Sample dimensions (L = 3.08 cm, W= 2.1 cm, T = 0.5 – 4.06 mm), 40 °C					
		0.5 mm	0.7 mm	1.18 mm	2.06 mm	3.02 mm	4.06 mm
Effective diffusivity	$D\ (\frac{cm^2}{min})$	1.11×10^{-3}	1.82×10^{-4}	7.77×10^{-4}	7.40×10^{-4}	7.74×10^{-4}	1.59×10^{-3}
Desorption rate	$k_s\ (min^{-1})$	4.32×10^{-2}	3.71×10^{-2}	4.22×10^{-2}	3.35×10^{-2}	3.24×10^{-2}	5.02×10^{-2}
Langmuir capacity	$C'_H\ (mg\ g^{-1})$	6.61×10^{-2}	6.02×10^{-2}	6.42×10^{-2}	4.90×10^{-2}	7.05×10^{-2}	6.82×10^{-2}
Langmuir affinity	$b'\ (mg^{-1}g)$	1.50×10^{1}	1.63×10^{1}	1.70×10^{1}	1.79×10^{1}	1.82×10^{1}	1.50×10^{1}
Pooling factor	$\alpha\ (mg\ g^{-1})$	1.30×10^{0}	2.22×10^{0}	2.13×10^{0}	1.55×10^{0}	2.43×10^{0}	3.0×10^{0}
Pooling threshold	$C_H^o\ (mg\ g^{-1})$	5.27×10^{-1}	3.17×10^{-1}	2.43×10^{-1}	1.73×10^{-1}	1.21×10^{-1}	3.61×10^{-1}
Pooling power	n (-)	1.27×10^{0}	2.0×10^{0}	1.41×10^{0}	2.31×10^{0}	2.06×10^{0}	2.08×10^{0}
Herny's law constant	$k_d(cm^{-3}\ g^{-1})$	1.40×10^{1}	1.43×10^{1}	1.49×10^{1}	1.70×10^{1}	1.48×10^{1}	1.50×10^{1}

S2. SCE optimized parameters for M9787 samples

Parameters	Symbol	Sample dimensions (L = 1.99 cm, W= 1.94 cm, T = 0.1847 cm)		
		30 ℃	40 ℃	50 ℃
Effective diffusivity	D	5.18×10^{-5}	5.368×10^{-5}	1.1×10^{-4}
Desorption rate	k_s	1.368×10^{-1}	9.99×10^{-1}	0.281×10^{1}
Langmuir capacity	C'_H	0.212×10^{1}	0.232×10^{1}	0.233×10^{1}
Langmuir affinity	b'	2.0×10^{1}	1.705×10^{1}	1.34×10^{1}
Pooling factor	α	0.737×10^{1}	0.773×10^{1}	0.654×10^{1}
Pooling threshold	C_H^o	2.29×10^{-2}	3.72×10^{-2}	6.45×10^{-2}
Pooling power	n	1.05	1.237	1.01
Herny's law constant	k_d	3.04×10^{1}	1.63×10^{1}	1.102×10^{1}
Reduced tortuosity	τ	6.36×10^{-1}	9.9×10^{-1}	6.48×10^{-1}

S3. SCE optimized parameters for Zircar RS-1200 samples

Parameters	Symbol	Sample dimensions (L = 2.65 cm, W= 1.074 cm, T = 0.327 cm)
		40 ℃
Effective diffusivity	D	1.0×10^{-2}
Desorption rate	k_s	0.91×10^{1}
Langmuir capacity	C'_H	0.332×10^{1}
Langmuir affinity	b'	0.472×10^{1}
Pooling factor	α	0.299×10^{1}
Pooling threshold	C_H^o	1.23×10^{-2}
Pooling power	n	1.05
Herny's law constant	k_d	4.77×10^{1}
Reduced tortuosity	τ	1.27×10^{-1}

S4. Dimensions of various 1D and 3D samples

Material	1D samples	3D samples
Sylgard-184	L = 3.08 cm, W= 2.1 cm, T = 0.5 - 4.06 mm	**Cylinder:** radius = 0.64 cm, height = 1.15 cm
M9787	L = 1.99 cm, W = 1.94 cm, and T = 0.185 cm	**Slab:** length = 1.95 cm, width = 1.93 cm, and thickness = 0.42 cm
Zircar RS-1200	L = 2.65 cm, W= 1.074 cm, T = 0.327 cm	**Slab**: length = 1.51 cm, width = 1.212, and thickness = 0.56 cm<br>**Cylinder**: radius = 0.5 cm, height = 1.0 cm

S5. Measurement uncertainties in experiments

The measurement uncertainties include relative humidity (RH) accuracy of ±1% (0 – 90%) with regulation accuracy of ±0.1% RH, temperature measurement accuracy of ±0.1 °C with regulation accuracy of ±0.1 °C, and weight resolution of ±1 µg (maximum sample size of 5 g). Mass calibration (for microbalance) is performed using certified standards of various masses, where uncertainty of ± 0.05 mg using a 100 mg calibration weight; ± 0.04 mg using a 50 mg calibration weight; ± 0.03 mg using a 20 mg calibration weight is expected. Due to stringent calibration and certification of microbalance, the uncertainty is not significant for practical purposes. We have tested the reproducibility of the data by performing experiments multiple times under the same conditions (T/RH). We note that the data acquired from the instrument are reproducible, and the error margin is less than 1% at max. Three sets of data obtained by running cyclic humidity steps experiment using a Zircar RS-1200 sample at 30 °C are shown in Fig. S1. Further evaluations of error margin are shown in Figures S2 and S3.

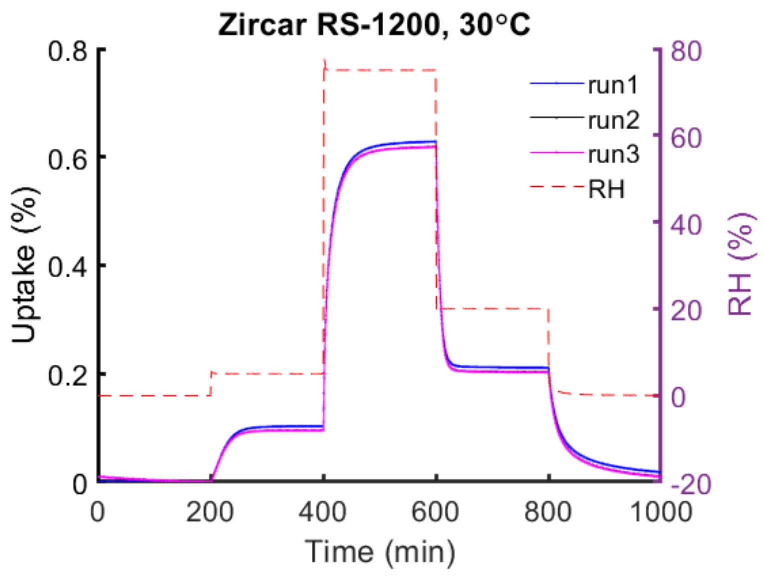

Figure S1: Moisture sorption by Zircar RS-1200 sample with relative humidity (RH) steps (i.e., 0% – 5% – 75% – 20% – 0 %) at 30 °C. The sample was exposed to the same conditions for three cycles.

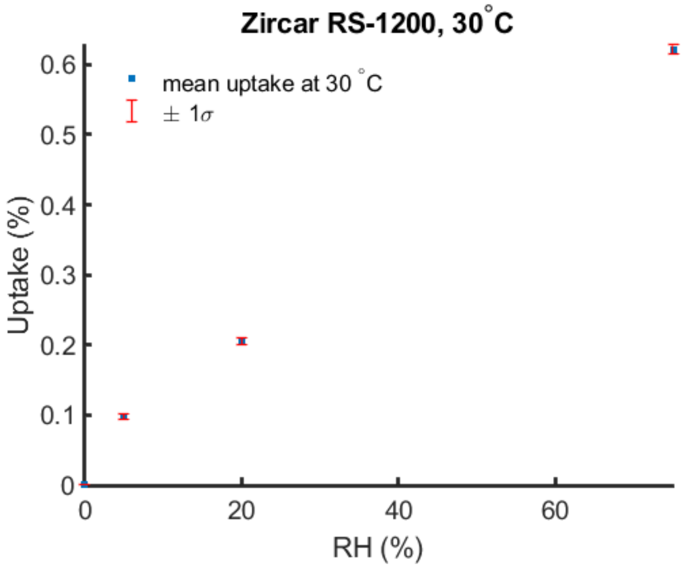

Figure S2: Equilibrium moisture sorption (from Fig. 1) by Zircar RS-1200 sample at each relative humidity (RH) steps at 30 °C. One standard deviation (±1σ) on each data point is plotted.

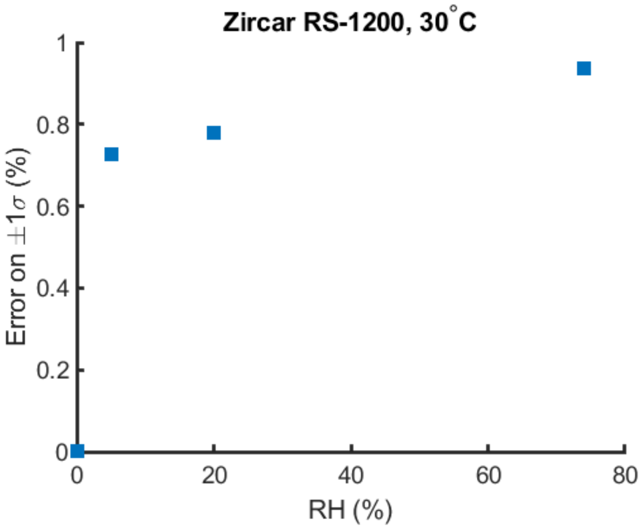

Figure S3: Percentage error computed using one standard deviation (±1σ) on each data point and the maximum uptake at 75% RH (i.e., $error = \frac{|1\sigma|}{\max uptake} * 100\ \%$). The uncertainty on each data point was <1%.

Next, we have verified that the moisture uptake level by a material during the typical isotherm experiment and while exposed to the same condition for a longer period. Our results show that the uptake level reached during the experiment guided an asymptote level is consistent with an

uptake level obtained by exposing the sample to the same condition for a long time. The results are shown in Figures S4 and S5.

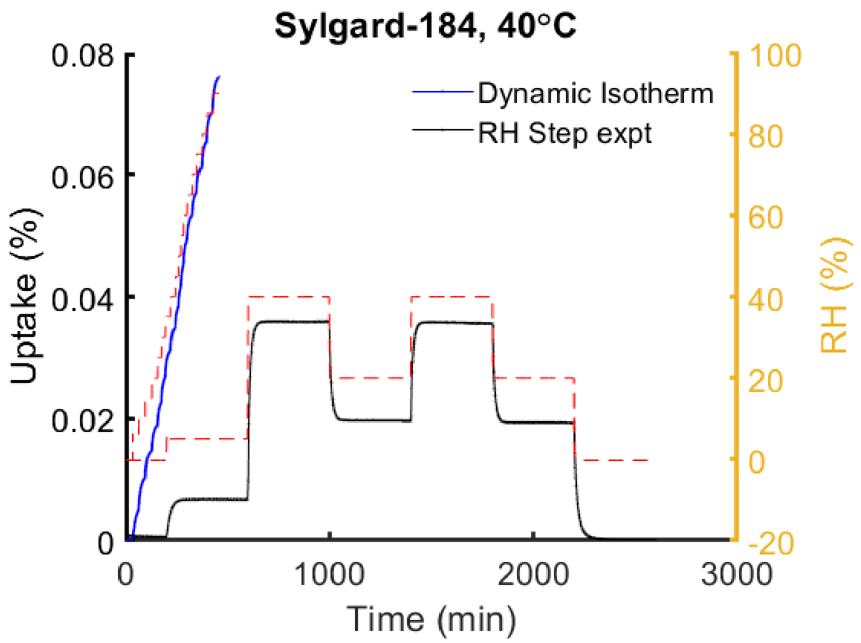

Figure S4: Moisture uptake profile of Sylgard-184 during dynamic isotherm and RH-step experiments. RH profiles are plotted on the right y-axis.

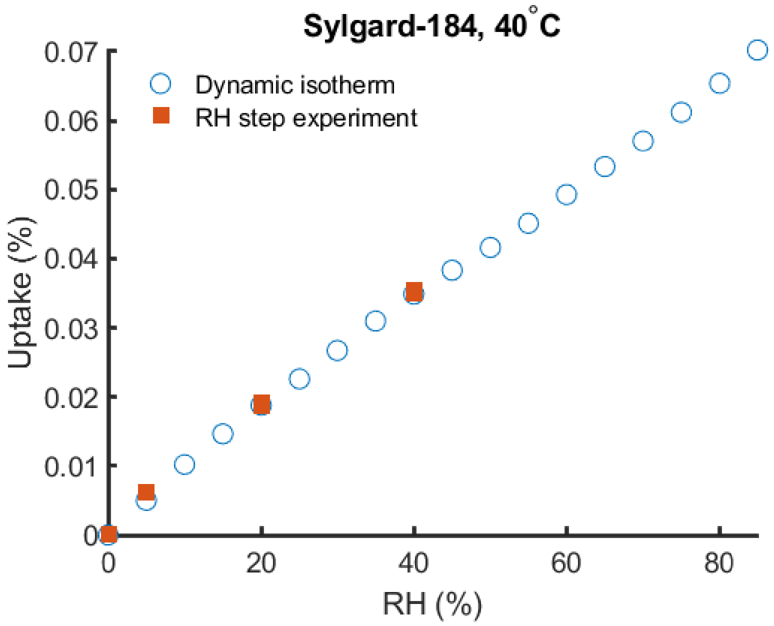

Figure S5: Maximum uptake (in wt%) vs. RH (%) plot obtained from Sylgard-184 data in Fig. 4. The uptake level is consistent between two different types of experiments. Results show that the pre-programmed isotherm experiments reached the maximum uptake level before stepping to the next RH level.
